# De Garengeot Hernia in a Male Patient: Case Report of an Incarcerated Appendix Managed by a Hybrid Approach

**DOI:** 10.70352/scrj.cr.26-0195

**Published:** 2026-06-03

**Authors:** Abdul Raheem Malik, Hassan Malik

**Affiliations:** 1 Department of Surgery, Continental Medical College, Lahore, Punjab, Pakistan; 2 Department of Surgery, Gold Coast University Hospital, Gold Coast, Queensland, Australia

**Keywords:** De Garengeot hernia, femoral hernia, case report, laparoscopic cecectomy, hybrid surgical approach

## Abstract

**INTRODUCTION:**

De Garengeot hernia is a rare condition in which the appendix is trapped within a femoral hernia. It accounts for less than 1% of femoral hernia cases and is more common in females, making its occurrence in males particularly unusual. Diagnosis is challenging because symptoms and laboratory findings are often nonspecific, and no standardized surgical protocol exists.

**CASE PRESENTATION:**

We report the case of a 69-year-old male who presented with a 4-day history of a tender, irreducible right groin lump. He was afebrile with stable vital signs and had unremarkable laboratory investigations. CT revealed the appendix within the right femoral hernia sac without signs of acute appendicitis. Laparoscopic exploration demonstrated the appendix tip within the femoral sac, with a bulky and gangrenous mesoappendix. A laparoscopic cecectomy was performed, followed by a low groin incision to reduce and excise the hernia sac. The femoral canal was narrowed with interrupted polypropylene sutures. The patient recovered uneventfully and was discharged the following day.

**CONCLUSIONS:**

This case highlights the diagnostic and therapeutic challenges of De Garengeot hernia and highlights the value of ongoing documentation to inform future surgical practice.

## INTRODUCTION

De Garengeot hernia is a rare condition in which the appendix is trapped within a femoral hernia. It was first described in 1731 by the Parisian surgeon, René Jacques Croissant de Garengeot, after whom it is named.^1)^ Overall, it represents only about 0.9% of femoral hernia cases and poses various diagnostic and therapeutic challenges.^2)^ Furthermore, it occurs predominantly in females, making cases in males especially uncommon.^3)^ This hernia should not be confused with Amyand’s hernia, in which the appendix is found inside an inguinal hernia.

Owing to the rarity of the phenomenon, there is limited literature available, with only 152 published papers since 1898^4)^ and fewer than 450 total cases reported.^5)^ Furthermore, a meta-analysis by Linder et al. concluded that there is no standard surgical approach to the problem and that diagnosis is not clarified by any specific laboratory or radiological investigations.^6)^

We present the case of a male patient with a De Garengeot hernia in whom the appendix and mesoappendix were found within the femoral hernia sac and were complicated by gangrenous changes. This case report has been prepared in line with the SCARE checklist.^7)^

## CASE PRESENTATION

**Fig. 1:**
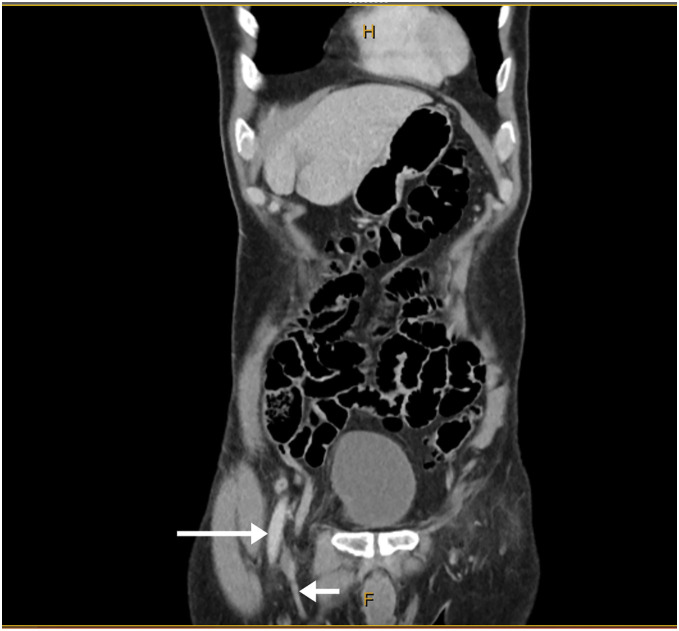
Coronal CT scan showing the appendix within the right femoral canal. The long arrow indicates the femoral artery and the short arrow indicates the femoral vein.

**Fig. 2:**
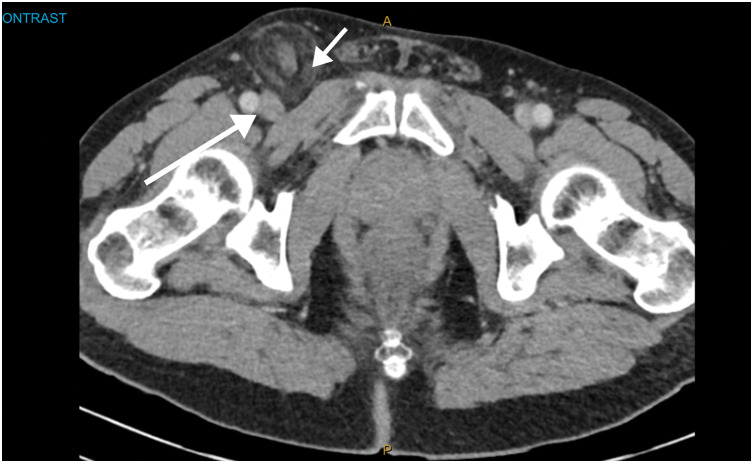
Axial CT confirming the appendix within the right femoral canal. The long arrow indicates the femoral vein, with the femoral artery lateral to it. The short arrow indicates the femoral hernia located below the inguinal ligament.

The patient was a 69-year-old male who presented with a 4-day history of a tender, irreducible right groin lump. He had no significant past medical or surgical history, no family history of hernias, took no regular medications, and was a non-smoker. He was admitted via the emergency department of the Gold Coast University Hospital. The patient was afebrile, with stable vital signs and unremarkable laboratory findings. CT demonstrated the appendix within the right femoral hernia sac without evidence of acute appendicitis (**Figs. 1** and **2**).

**Fig. 3:**
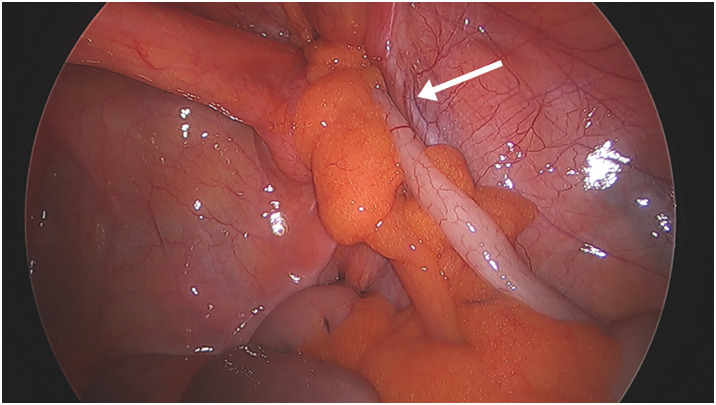
Laparoscopic view showing the appendix within the femoral hernia below the inguinal ligament. The arrow indicates the femoral vein.

**Fig. 4:**
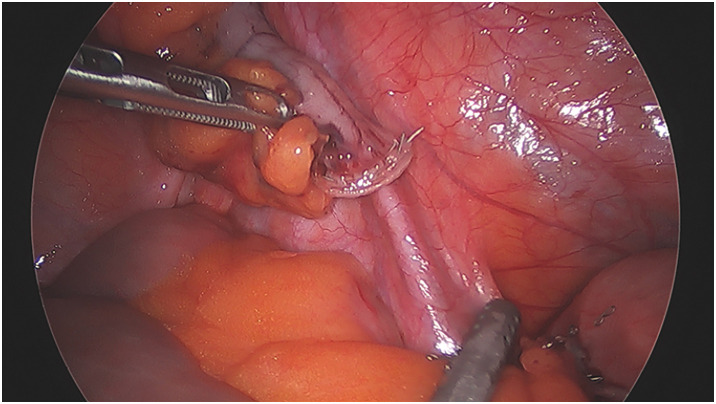
Laparoscopic view showing the divided base of the appendix after cecectomy.

The patient underwent laparoscopic exploration via a trans-umbilical approach. Pneumoperitoneum was established using the open Hasson technique with a balloon port, and 5-mm ports were placed in the suprapubic and left iliac fossa positions under direct vision. Intravenous antibiotics were administered at induction. Intraoperatively, the tip of the appendix was identified within the right femoral hernia sac and could not be reduced, even with external pressure applied at the femoral ring. The mesoappendix was congested and dark purple, with patchy areas of necrosis. The femoral sac was dissected bluntly away from the femoral vein, and its neck was clearly visualized beneath the inguinal ligament. Tissue viability was assessed visually and by gentle needle prick to check for bleeding before proceeding with resection. The hernia sac was confirmed to be femoral in origin, located inferior to the inguinal ligament and medial to the femoral vein. These anatomical landmarks were visualized laparoscopically and confirmed during open exploration (**Fig. 3**). The appendix tip itself appeared non-inflamed; however, the mesoappendix at the tip was bulky and gangrenous. A laparoscopic cecectomy was performed, with the mesoappendix secured at the base using a LigaSure Atlas tissue fusion laparoscopic instrument (Medtronic, Minneapolis, MN, USA) (**Fig. 4**). Normally, appendectomy proceeds in a tip-down retrograde fashion; however, the incarcerated and irreducible appendix tip prevented this approach entirely. Any anterograde dissection from the base toward the tip risked tearing the gangrenous, friable mesoappendix, with consequent intraperitoneal soiling and uncontrolled hemorrhage. A stapled limited cecectomy was therefore undertaken, allowing division through a well-vascularized and unaffected segment of cecal wall at a safe distance from the compromised tissue, securing both hemostatic control and a clear resection margin without instrumenting the necrotic mesoappendix.

A separate low right groin incision (low femoral hernia approach) was then made to access the femoral hernia. The sac was opened and reduced intraperitoneally, followed by excision of the gangrenous sac, which was sent for histopathology. The appendix was retrieved in an endobag. The femoral canal was narrowed with interrupted 2-0 PROLENE Polypropylene sutures (ETHICON, Raritan, NJ, USA) between the inguinal and pectineal ligaments, leaving adequate space for venous return. The procedure was performed by a consultant general surgeon experienced in advanced laparoscopic hernia repair. The intraoperative time was recorded as 70 min, and the blood loss was estimated to be 10 mL.

**Fig. 5:**
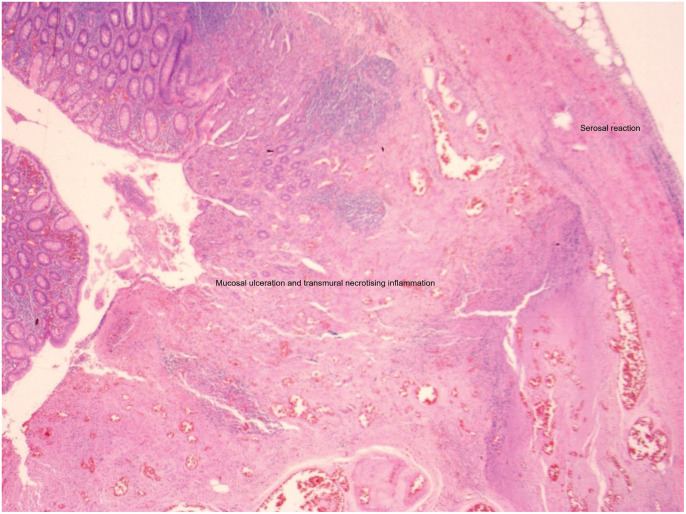
Hematoxylin and eosin-stained section of the resected specimen. The image demonstrates mucosal ulceration, transmural necrotizing inflammation, and serosal reaction.

The patient had an uneventful recovery, tolerated a normal diet postoperatively, and was discharged home the following day with oral analgesia. Follow-up in the clinic was arranged for 2 weeks to review the wounds and histology. The patient remained well at follow-up with no complications. Microscopic examination of the hernia sac demonstrated fibrinoid necrosis and acute inflammatory infiltration. Histological sections of the resected specimen confirmed transmural necrotizing inflammation with mucosal ulceration and serosal reaction, in keeping with incarceration-mediated ischemia (**Fig. 5**).

## DISCUSSION

Femoral hernias account for 3%–5% of groin hernias, and De Garengeot hernias make up less than 1% of those cases.^8)^ Moreover, the incidence of De Garengeot hernia is much more common in post-menopausal women than in men,^3)^ with only 17 male cases being included in a 2022 systematic review.^4)^ In our case, the patient was male, which makes this presentation particularly rare.

The presence of the appendix in a femoral hernia is a rare and challenging clinical scenario. Clinically, De Garengeot hernia often mimics an incarcerated femoral hernia, with nonspecific symptoms and often unremarkable laboratory results, as in our case. The absence of elevated inflammatory markers despite a gangrenous mesoappendix is consistent with incarceration-mediated ischemia, in which segmental vascular compromise may precede systemic inflammatory activation; this is pathophysiologically distinct from primary appendicitis. The sensitivity of CT for diagnosing this condition preoperatively is limited, and it is even lower for US.^9)^ Nevertheless, CT remains the best imaging modality when employed,^10)^ and its use was critical in our patient’s preoperative assessment and surgical planning. In our patient, the key CT features enabling preoperative diagnosis were the identification of a blind-ending tubular cecal outgrowth within the femoral hernia sac, positioned medial to the femoral vein and below the inguinal ligament, distinguishing it from Amyand’s hernia, in which the appendix occupies an inguinal sac above the inguinal ligament. The absence of periappendiceal fat stranding or mural thickening confirmed that the appendix was not primarily inflamed, and the blind-ending morphology differentiated it from the incarcerated small bowel.

The predominance of De Garengeot hernia in post-menopausal women likely reflects the anatomically wider female pelvis, a broader femoral canal, and ligamentous laxity associated with parity and estrogen deficiency. In males, the femoral canal is inherently narrower, substantially reducing the risk of both femoral herniation and entrapment of the appendix. The occurrence in our male patient may be attributable to idiosyncratic anatomical factors, including a congenitally widened femoral ring or an unusually mobile appendix with a mesoappendix of sufficient length to migrate into the femoral sac.

No standardized protocol exists for managing these types of cases, and decisions must be individualized based on intraoperative findings. Traditional approaches include an open groin incision with appendectomy and herniorrhaphy. Minimally invasive alternatives, including laparoscopic and even staged robotic approaches, have been explored in recent years.^11,12)^ In our case, a hybrid approach was employed, with a laparoscopic cecectomy combined with open femoral repair. This allowed comprehensive management of both the intra-abdominal pathology and the hernia defect. Because our patient had gangrenous changes in the tip of the mesoappendix due to strangulation at the femoral ring, mesh repair was avoided. Instead, the femoral canal was narrowed using a suture repair between the inguinal and pectineal ligaments, while the surgical view was maintained by shining the laparoscopic light intra-abdominally to define the proximity of the femoral vein and prevent narrowing. A fully laparoscopic hernia repair was not pursued because the gangrenous contamination prevented mesh placement; instead, suture repair of the femoral canal was performed more reliably under direct vision through the groin incision. Appendectomy via a groin incision alone would have provided inadequate intra-abdominal visualization and would have prevented safe controlled division of the cecal base.

This report has several limitations. The 2-week follow-up period is insufficient to evaluate long-term hernia recurrence. While suture repair of the femoral canal was appropriate given the contaminated field, it carries a higher theoretical recurrence risk that mesh-based repair; longer-term follow-up is therefore warranted. As with all case reports, findings from a single case cannot be generalized, and registry-based data will be necessary to establish optimal surgical strategies for this rare condition.

## CONCLUSIONS

De Garengeot hernia remains a rare surgical entity, especially in male patients. This type of femoral hernia is seldom diagnosed preoperatively and has no standard surgical protocol. Surgical management should be individualized, with the choice of appendectomy, cecectomy, and method of hernia repair guided by intraoperative findings and the degree of contamination. Given its rarity and the limited number of reported cases, continued documentation of these hernias is essential to improve understanding of their presentation, variations in pathology, and optimal surgical strategies.
